# Effects of neuromuscular training (NEMEX-TJR) on patient-reported outcomes and physical function in severe primary hip or knee osteoarthritis: a controlled before-and-after study

**DOI:** 10.1186/1471-2474-14-232

**Published:** 2013-08-08

**Authors:** Eva Ageberg, Anna Nilsdotter, Eva Kosek, Ewa M Roos

**Affiliations:** 1 Department of Health Sciences, Lund University, PO Box 157, Lund, SE-221 00, Sweden; 2 R&D Department, Central Hospital, Halmstad, Sweden; 3 Osher Center for Integrative Medicine, Department of Clinical Neuroscience, Karolinska Institute, Stockholm, Sweden; 4 Institute of Sports Science and Clinical Biomechanics, University of Southern Denmark, Odense, Denmark

**Keywords:** Osteoarthritis, Arthroplasty, Exercise therapy, Patient-reported outcomes, Performance-based measures

## Abstract

**Background:**

The benefits of exercise in mild and moderate knee or hip osteoarthritis (OA) are apparent, but the evidence in severe OA is less clear. We recently reported that neuromuscular training was well tolerated and feasible in patients with severe primary hip or knee OA. The aims of this controlled before-and-after study were to compare baseline status to an age-matched population-based reference group and to examine the effects of neuromuscular training on patient-reported outcomes and physical function in patients with severe primary OA of the hip or knee.

**Methods:**

87 patients (60–77 years) with severe primary OA of the hip (n = 38, 55% women) or knee (n = 49, 59% women) awaiting total joint replacement (TJR) had supervised, neuromuscular training (NEMEX-TJR) in groups with individualized level and progression of training. A reference group (n = 43, 53% women) was included for comparison with patients’ data. Assessments included self-reported outcomes (HOOS/KOOS) and measures of physical function (chair stands, number of knee bends/30 sec, knee extensor strength, 20-meter walk test) at baseline and at follow-up before TJR. Analysis of covariance (ANCOVA) was used for comparing patients and references and elucidating influence of demographic factors on change. The paired *t*-test was used for comparisons within groups.

**Results:**

At baseline, patients reported worse scores than the references in all HOOS/KOOS subscales (hip 27–47%, knee 14–52%, of reference scores, respectively) and had functional limitations (hip 72–85%, knee 42–85%, of references scores, respectively). NEMEX-TJR (mean 12 weeks (SD 5.6) of training) improved self-reported outcomes (hip 9–29%, knee 7–20%) and physical function (hip 3–18%, knee 5–19%) (p < 0.005). Between 42% and 62% of hip OA patients, and 39% and 61% of knee OA patients, displayed a clinically meaningful improvement (≥15%) in HOOS/KOOS subscales by training. The improvement in HOOS/KOOS subscale ADL was greater for patients with knee OA than hip OA, while the improvement in subscale Sport/Rec was greater for patients with hip OA than knee OA.

**Conclusions:**

Both self-reported outcomes and physical function were clearly worse compared with the reference group. Neuromuscular training with an individualized approach and gradual progression showed promise for improving patient-reported outcomes and physical function even in older patients with severe primary OA of the hip or knee.

## Background

There is to date high level evidence supporting the benefits of exercise in mild and moderate knee or hip OA [1], but the evidence in severe OA is less clear [2]. Studying effects of exercise at this late stage of the disease can include people awaiting total joint replacement (TJR) of the hip or knee as they have severe OA.

A recent meta-analysis [3], show low to moderate evidence that exercise before TJR reduces pain in patients with hip or knee OA, and improves patient-reported function in those with hip OA. However, due to insufficient reporting, few studies were included and most trials included a small number of participants [3], implying a need for further studies. Only a limited number of studies included both patient-reported outcomes and measures of physical function [3], however, several authors now suggest that both measurement properties be used to obtain a complete picture of function in patients with OA [4-6]. The training programs included traditional exercise therapies for people with hip or knee OA, such as aerobic exercise and/or strengthening training targeting muscle weakness [3].

Besides muscle weakness, patients with OA have impaired sensorimotor function, in terms of sensory deficiency [7,8], altered muscle activation patterns [9], and reduced functional performance [10]. From this perspective, it seems apparent that training programs should address several aspects of sensorimotor function to improve function and alleviate symptoms. Neuromuscular training may meet these needs. While strength training aims primarily at increasing motor output, neuromuscular training aims principally at improving quality and efficiency of movements. In young and middle-aged with knee injuries, at high risk of knee OA [11], such training programs were effective in improving function and reducing symptoms [12-17].

Recently, we applied these principles of neuromuscular training to people with severe hip or knee OA, and reported that an individualized neuromuscular training program (NEMEX-TJR), was well tolerated and feasible in these patients, in terms of no worsening of pain, few joint-specific adverse events, and achieved progression of training level [18].

The aims of this prospective cohort study were: 1) to compare baseline status to an age-matched population-based reference group; and 2) to examine the potential effects of the NEMEX-TJR on patient-reported outcomes and physical function in patients with severe primary OA of the hip or knee waiting for TJR. We hypothesized that patients had functional impairments at baseline and that NEMEX-TJR would improve patient-reported outcomes and physical function at follow-up before surgery compared with the baseline assessment.

## Methods

### Trial design

A controlled before-and-after study which conforms to the TREND statement for non-randomized study designs, with a normative reference group.

### Patients

130 patients between 60 and 77 years old with severe primary OA of the hip (n = 51, 55% women) or knee (n = 83, 60% women), all assigned for TJR, were recruited from the Department of Orthopedics, Skåne University Hospital Lund, Sweden, during 2007–2009 (Table 1). Severe OA was defined as being eligible for TJR. Those eligible for TJR constitute a small fraction of all patients with joint pain [19] and TJR eligibility criteria include pain, severe disability, and radiographic disease [20]. Exclusion criteria were post traumatic OA (e.g., fracture), rheumatoid arthritis, psoriatic arthritis, congenital hip deformities, Perthes’ disease, THR or TKR during the last 12 months, severe heart failure or neurological diseases affecting physical function, dementia, and not Swedish-speaking due to the high level of language skills required for questionnaires and assessment. Patients treated with antidepressive, neuroleptics, anticonvulsive drugs, or cortisone, were also excluded.

**Table 1:** Characteristics of the patients at baseline

**Characteristic**		**Total OA group (n = 87)**	**Hip OA (n = 38)**	**Knee OA (n = 49)**	**References (n = 43)**
Women (n (%))		50(58)	21(55)	29(59)	23(54)
Age (y), mean (SD)		68(4.1)	67(3.8)	69(4.2)	69(4.6)
BMI (kg/m^2^), mean (SD)		28.9(4.5)	27.8(4.3)	29.8(4.5)	26.5(3.4)
Index joint, right (n (%))		51(59)	19(50)	32(65)	NA
Analgesics					
	Regular use	63	33	30	2
	Sporadic	11	3	8	18
	No	13	2	11	23
Previous surgery (n)					
	Knee/hip	26	9	17	7
	Foot	16	6	10	5
	Back/neck	7	3	4	1
	Upper extremity	26	9	17	6
Co-morbidities* (n)					
	0	36	20	16	27
	1	38	15	23	8
	≥2	13	3	10	8
Considered TJR (mths), median (quartiles)		7(4–12)	7(4–12)	8(5–19)	NA
Daily activities (n)					
	Easy (mainly sitting)	40	17	23	19
	Somewhat hard (walking)	38	17	21	21
	Hard/very hard (walking, lifting)	9	4	5	3
Physical therapy training for hip/knee within last year					
	No	64	29	35	41
	Yes	23	9	14	2
Walking aid					
	No	80	33	47	43
	Yes	7	5	2	0
Marital status					
	Married/cohabiting	60	26	34	34
	Divorced	11	5	6	4
	Widowed	12	5	7	2
	Single	3	2	1	3
Immigrant (n)		8	5	3	7
Education					
	Elementary school	55	24	31	16
	High school	9	6	3	15
	College or University	19	7	12	11
	Other	4	1	3	1
Smoking					
	No	49	20	29	25
	Previously	33	14	19	16
	Yes	5	4	1	2
Alcohol consumption					
	Never	6	3	3	3
	Rarely	16	5	11	6
	Monthly	30	13	17	8
	Weekly	34	16	18	22
	Daily	1	1	0	4

* Back, lung, hypertension, heart, peripheral arteries, neurological, diabetes, cancer, ulcer, kidney, vision.

All patients were offered supervised NEMEX-TJR training in groups. Forty patients declined to participate in the group training because they had an established contact with a physical therapist at their local health care center, or because of problems with transportation to the intervention site. These patients were younger than those who agreed to participate in group training (mean difference −2.4 years, 95% confidence interval (CI) -3.96;-0.87). There were no differences between the nonparticipants and the participants in gender (60% vs 59% women, p = 0.848), index joint (33% vs 42% hip OA, p = 0.335), BMI (mean difference 1.30 95% CI −0.38;2.97), or in baseline scores for HOOS/KOOS pain (mean difference −3.8 95% CI −8.7;1.0), symptoms (mean difference −1.7 95% CI −7.9;4.6), ADL (mean difference −3.5 95% CI −8.9;1.9), sport/rec (mean difference −2.0 95% CI −8.1;3.5), or QOL (mean difference −2.0 95% CI −7.1;3.0).

At the follow-up assessment, 3 patients (3%, 1 women) with knee OA withdrew from the study for the following reasons: declined surgery and follow-up (n = 1), and unknown reason (n = 2). Data presented are from the 87 patients (hip OA, n = 38; knee OA, n = 49) who completed the baseline and follow-up assessments. Seventy-six of these patients were included in a previous report on feasibility of neuromuscular training [18].

### Reference group

A reference group was included for comparison with patients’ baseline data and to assess any systematic change in outcomes. A random sample from the population, identified through the Swedish civil registration system, were recruited during 2007–2009 and constituted the reference group. We aimed at including 40 references. To account for about 50% non-responders, comparable with other population-based cohorts [21], and approximately 20% not meeting the inclusion criteria, an invitation was sent by mail to 141 people between 60 and 75 years old living in the same geographic area as the patients. Those who did not reply within 2 weeks were contacted by telephone. 42 did not respond to mail or phone call, and 27 declined to participate (49%). People who accepted the invitation were assessed for eligibility by telephone (n = 72). Subjects that had been treated for hip or knee disorders within the last year (n = 11), or fulfilled any exclusion criteria (n = 16), as described for the patients, were excluded. 45 people accepted the invitation. Two of these withdrew because of medical issues unrelated to the study. The remaining 43 subjects (23 women) were included (Table 1). They were assessed twice, to determine any systematic change in outcomes, with a mean of 13 weeks (SD 1.2, range 9 to 17) between test sessions. The references did not undergo exercise intervention.

The research ethics committee at Lund University approved the study (LU 81/2006) and the participants signed a written informed consent form.

### Neuromuscular training method

The neuromuscular training method is based on biomechanical and neuromuscular principles and aims to improve sensorimotor control and achieve compensatory functional stability. Sensorimotor control is the ability to produce controlled movement through coordinated muscle activity, and functional stability (also called dynamic stability) is the ability of the joint to remain stable during physical activity [22]. The principles are described in detail previously [13-16,18]. In summary, the principles include: *Active movements in synergies* of all the joints in the injured extremity; applying *bilateral transfer effect of motor learning* to the injured leg by initiating the normal movement on the other leg; exercises mainly performed in *closed kinetic chains* to enhance proprioceptive information from the foot soles and to obtain *co-activation of stabilizing muscles*; enhancement of *postural functions* of weight bearing muscles; using voluntary movements in the other lower extremity, trunk and arms or unexpected movements to achieve *postural reactions* (feed-forward and feedback control) in the injured leg; and emphasizing *quality of performance* in each exercise with an appropriate position of the joints in relation to each other (postural orientation).

The goal is to obtain equilibrium of loaded segments in static and dynamic situations and acquire postural control in situations resembling conditions of daily life and more strenuous activities. Emphasis is put on efficiency and quality of movements of each exercise. Several aspects of sensorimotor function, such as strength, coordination, balance, and proprioception, are included in the exercises, but focus can be, e.g., balance in one exercise and strength in another. To achieve the desired requirement of postural activity, patients perform exercises in various positions, i.e., lying, sitting, and standing.

The training is individualized, because symptoms and functional limitations are heterogeneous in people with an injury or disease. The level of training and progression is guided by the patient’s sensorimotor function, taking into account various factors related to the individual (e.g., symptoms, age, gender, previous and target activity level) and the injury/disease (e.g., affected joint structures, type and severity of injury/disease). Progression is provided by; varying the number of, direction, and velocity of the movements; increasing the load; changing the support surface, and/or utilizing unexpected movements.

We have named the neuromuscular training method NEuroMuscular EXercise (NEMEX). A suffix is added to indicate the group of patients to which that program applies. In this particular study, the training program is called NEMEX-TJR, where TJR stands for Total Joint Replacement [18].

The principles of neuromuscular training are applied in the NEMEX-TJR training program as described [18]: The training sessions consist of three parts: warming up, a circuit program, and cooling down. The warm-up period consists of ergometer cycling for 10 minutes. The circuit program comprises four exercise circles, including exercises with the key elements: core stability/postural function; postural orientation; lower extremity muscle strength; and functional exercises. The goal of each exercise is to enhance appropriate muscle activation to obtain functional stabilization of joints, reduce joint load, and achieve quality and efficiency of movements and thereby optimize the patient’s function. The quality of the performance in each exercise with an appropriate position of the joints in relation to each other, i.e., with the hip, knee and foot well aligned (postural orientation), is emphasized. To allow for progression, three levels of difficulty are given for each exercise. Progression is made when an exercise is performed with good sensorimotor control and good quality of performance (based on visual inspection by the physical therapist) and with minimal exertion and control of the movement (perceived by the patient). The last part of the training program includes cooling down, and stretching exercises for the lower extremity muscles (10 minutes). The exercises in the NEMEX-TJR training program and their progression levels are described in the additional file in Ageberg et al. [18].

Training took place in groups at an exercise facility, under the supervision of an experienced physical therapist specializing in training of musculoskeletal disorders. Patients continuously entered the group training, i.e., the group held both novice patients and those who had participated in several training sessions and, thus, were more familiar with the training. Most often, about ten patients attended a training session. During each group training session, each participant was monitored individually so that the exercises were performed at a training level according to their sensorimotor function. The patients were offered 2 training sessions a week of 60 minutes each. The training sessions took place late morning/before noon, since patients with hip or knee OA often report more pain early morning and in the afternoon.

Because pain is a major symptom for patients with hip or knee OA, we included a scale for monitoring pain during training. The patients were told that pain was allowed up to 5 on a 0 to 10 scale during and after the training session [23]. They were also told that the day after training, pain should subside to “pain as usual”. If pain did not subside, the level of training was reduced [23]. This pain monitoring system is part of the NEMEX-TJR concept as described (available in additional file in Ageberg et al. [18]).

The patients participated in the training until they underwent TJR. The number of weeks of training was dependent on the waiting list for surgery, and was not pre-defined in the study. The mean (SD, range) number of weeks from baseline to follow-up, i.e., until surgery, was 15 (SD 7.1, range 4 to 46).

The physical therapist was not assessed for adherence with the NEMEX-TJR, but the fact that she contributed to the design of the training program likely enhanced adherence.

### Assessment

The patients and references performed the tests in the order that they are described below at baseline and at follow-up. An experienced assessor, who was well trained in all outcome measures from pilot-testing preceding the present study, performed the measurements.

#### Self-reported outcomes

##### HOOS/KOOS

The subjects rated their hip/knee and associated problems using the HOOS/KOOS [24,25]. The HOOS/KOOS are valid, reliable and responsive disease-specific self-administered questionnaires for patients assigned for THR/TKR [25,26]. Each questionnaire comprises five subscales; pain, other symptoms, activities in daily living (ADL), function in sport and recreation (Sport/Rec), and hip/knee related quality of life (QOL). Each subscale is scored on a 0 (worst) to 100 (best) scale. The corresponding HOOS/KOOS subscales were combined in the analyses.

#### Measures of physical function

##### Chair stands

The time required for 5 repetitions to rise from a chair and sit down was performed according to the OsteoArthritis Initiative (OAI) manual including detailed standardizations and instructions (available from: http://oai.epi-ucsf.org). The test requires lower extremity strength, balance, coordination, and flexibility. The participant sat on a straight-backed chair without arms, with seat height of 45 cm, and feet placed on the floor with knees flexed to slightly greater than 90 degrees. The participant wore comfortable shoes and kept the arms folded across the chest. The time it took to stand five times was recorded in number of seconds, to a hundredth of a second. A lower value indicates better performance. The test was performed twice, and the best (lowest) value was used in the analysis. Moderate agreement (coefficient of variation 13.9%) and excellent reliability (intraclass correlation coefficient 0.89) was reported in patients with severe hip or knee OA (mean 69, SD 7.2 years) [27].

### Number of knee bendings per 30 sec

Maximum number of knee bendings in 30 seconds was performed as described [28]. The test aims at evaluating muscle endurance and fast changes between eccentric and concentric muscle force over the knee joint. The subject stood with the long axis of the foot aligned to the stem of a “T” marked with tape on the floor. Finger tip support for balance was provided by the examiner. The participant was asked to look down and bend the knee, without bending forward from the hip, until he/she no longer could see the line along the toes (corresponding to about 50 degrees of knee flexion), and then return to extension. 10 seconds practice preceded the measurement. The test was performed on both legs, starting with the right leg. The number of knee-bendings performed in 30 s was recorded for the affected and non-affected legs, respectively [28]. A higher value indicates better performance. Moderate agreement (coefficient of variation 13.2%) and good reliability (intraclass correlation coefficient, 0.80) was reported in patients with severe hip or knee OA [27].

### Knee extensor strength

A hand-held dynamometer (Baseline® evaluation instruments, White Plains, USA) was used for assessing isometric knee extension strength in a sitting position with hips and knees flexed 90 degrees and hands resting in lap [29]. The dynamometer was fixed to the chair. Three measurements were taken, each trial lasting 7 seconds, with 1 minute rest between trials. The test was performed on both legs, starting with the right leg. The best value (in kg) for the affected and non-affected legs, respectively, was used in the analysis. A higher value indicates better performance. Because the validity of hand-held dynamometry can be questioned, such as underestimation of absolute quadriceps strength compared with the Biodex [30], the absolute values of quadriceps strength in the present study are not to be used for comparison with other cohorts.

### 20-meter walk test

The time required for the participant to walk 20 meters at their usual walking pace, and the number of steps that they took to walk this distance, was assessed according to the OsteoArthritis Initiative (OAI) manual including detailed standardizations and instructions (available from: http://oai.epi-ucsf.org). The participant walks 20 meters in one direction and then repeats the 20-meter walk by walking back in the other direction. The number of steps taken, the time it took (in seconds, to a hundredth of a second), and whether or not a walking aid was used was recorded. A lower value indicates better performance. The test was performed twice, and the best (lowest) value was used in the analysis. Good agreement (coefficient of variation 4.3%) and excellent reliability (intraclass correlation coefficient, 0.93) was reported in patients with severe hip or knee OA [27].

### Statistical analysis

The paired *t*-test was used for comparisons within groups. Analysis of covariance (ANCOVA), adjusted for baseline HOOS/KOOS pain and baseline value for the respective outcome, was used to elucidate the influence of demographic factors on change in outcomes in the following model: joint (hip/knee), sex, age, BMI, time the patient had considered TJR, and participation in supervised or home based physical therapy training within last year. Relative improvement was calculated by dividing the change between baseline and preop by the baseline value and multiplying by 100. An improvement ≥15% for an individual in the HOOS/KOOS was used to determine responders by treatment [31,32]. ANCOVA adjusted for age, sex, and BMI, was used for comparing baseline values between patients and references. Because there were no differences between right and left legs in the single-leg tests for the references, the right leg was used. To illustrate patients’ data relative to reference data, each patient’s score for the respective variables were normalized for the corresponding mean reference score. A level of p ≤ 0.05 was chosen to indicate statistical significance.

## Results

### Patients vs. references

**Figure 1:**
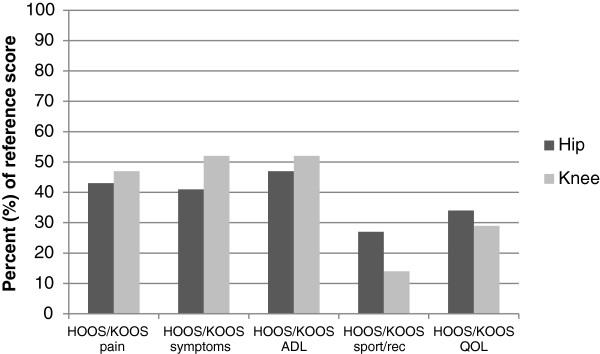
**Patients’ scores for HOOS/KOOS subscales relative to the reference scores (%) at baseline.** 100% represents the score of the reference population.

**Figure 2:**
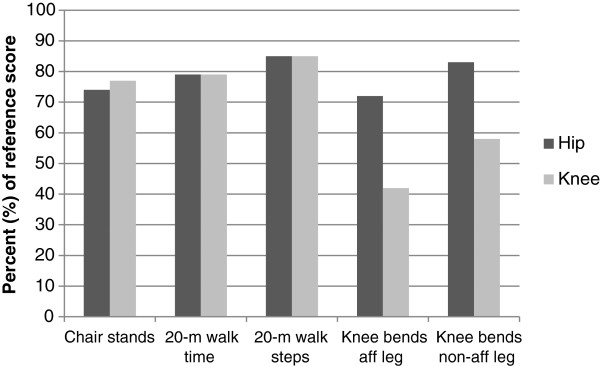
**Patients’ scores for performance-based measures relative to the reference scores (%) at baseline.** 100% represents the score of the reference population. Aff = affected, non-aff = non-affected.

Patients reported worse scores in all HOOS/KOOS subscales compared with the references at baseline. They also exhibited poorer physical function, except for knee extensor strength for the non-affected leg (Table 2). In general, there were larger discrepancies between patients and references for self-reported outcomes (hip OA between 27% and 47% of reference scores, knee OA between 14% and 52% of reference scores) than for physical function measures (hip OA between 72% and 85% of reference scores, knee OA between 42% and 85% of reference scores) (Figures 1 and 2).

**Table 2:** Scores for patient-reported outcomes and physical function measures and differences between patients and references at baseline

		**Total OA group**	**Hip OA**	**Knee OA**	**References**	**Total OA group**	**Hip OA**	**Knee OA**
		**(n = 83 to 87)**	**(n = 37 to 38)**	**(n = 45 to 49)**	**(n = 42 to 43)**	vs. references*	vs. references*	vs. references*
		**Mean (SD)**	**Mean (SD)**	**Mean (SD)**	**Mean (SD)**	**Mean diff (95% CI)**	**Mean diff (95% CI)**	**Mean diff 95% CI)**
**Patient-reported outcomes**								
HOOS/KOOS subscales								
	Pain	42(12.5)	40(12.5)	43(12.4)	90(14.4)	−47(−52.6;-42.4)	−50(−55.7;-43.8)	−45(−51.1;-39.5)
	Symptoms	42(15.9)	38(15.4)	46(15.4)	88(14.3)	−46(−51.3;-39.8)	−49(−56.1;-42.8)	−42(−48.5;-35.5)
	ADL	46(13.1)	44(12.9)	48(13.2)	91(13.1)	−43(−48.2;-38.3)	−46(−51.6;-40.2)	−41(−46.5;-35.4)
	Sport/Rec	17(14.9)	23(15.4)	12(12.5)	83(22.6)	−64(−70.8;-57.4)	−60(−67.2;-52.0)	−69(−76.4;-61.1)
	QOL	27(13.6)	29(14.9)	26(12.3)	86(18.6)	−56(−62.0;-50.5)	−55(−61.9;-48.4)	−57(−63.9;-50.6)
**Physical function**								
Chair stands (sec)		14.60(5.76)	14.90(4.18)	14.34(6.78)	10.08(2.06)	4.06(2.23;5.88)	4.73(2.61;6.86)	3.43(1.34;5.52)
Knee bendings/30 sec affected leg (n)^‡^		13(6.9)	16(6.4)	11(6.6)	23(6.8)^†^	−9(−11.6;-6.5)	−8(−11.1;-5.1)	−10.3(−13.6;-7.0)
Knee bendings/30 sec non-affected leg (n)^‡^		16(7.7)	19(7.4)	13(7.1)		−6(−9.1;-3.9)	−5(−7.5;-1.5)	−8(−11.4;-5.4)
Knee ext affected leg (kg)		17.0(5.0)	17.9(5.0)	16.3(5.0)	20.0(6.2)^†^	−3.5(−5.2;-1.7)	−2.5(−4.6;-0.5)	−4.3(−6.2;-2.3)
Knee ext non-affected leg (kg)		19.0(5.1)	19.8(5.4)	18.4(4.8)		−1.3(−3.1;0.4)	−0.7(−2.7;1.4)	−1.9(−3.9;-0.0)
20-m walk test, time (sec)		18.91(5.07)	18.48(3.61)	19.25(6.00)	14.11(1.87)	4.07(2.53;5.62)	4.18(2.37;5.99)	3.98(2.20;5.76)
20-m walk test, steps (n)		33(6.4)	32(4.3)	33(7.7)	27(2.5)	4(2.6;6.3)	5(2.4;6.8)	4(2.2;6.4)

*Analysis of variance, adjusted for age, gender, and BMI.

^†^Right leg in references used for comparison with patients’ affected and non-affected legs.

^‡^Could not perform the test: Total OA group: affected leg n = 33, non-affected leg n = 15; Hip OA: affected leg n = 9, non-affected leg n = 4; Knee OA: affected leg n = 24, non-affected leg n = 11.

Ext = extension strength.

CI = confidence interval. Confidence intervals not containing zero indicate statistical significance.

### Systematic change in outcomes in the reference group

A systematic change in the mean was noted for chair stands and the number of knee bendings/30 seconds in the reference group, indicating a small learning effect (confidence intervals close to zero) (Table 3). As the systematic change had no influence on the results between patients and references, it was not adjusted for in the analysis.

**Table 3:** Change in outcome measures baseline vs. follow-up in patients and references

		**Change baseline vs follow-up**			
		**Total OA group**	**Hip OA**	**Knee OA**	**References**
		**(n = 83 to 87)**	**(n = 31 to 38)**	**(n = 45 to 49)**	**(n = 41 to 43)**
		**Mean diff (95% CI)**	**Mean diff (95% CI)**	**Mean diff (95% CI)**	**Mean diff (95% CI)**
**Patient-reported outcomes**					
HOOS/KOOS subscales					
	Pain	5.7(3.3;8.0)	6.1(2.9;9.3)	5.3(1.9;8.7)	0.5(−3.1;4.1)
	Symptoms	4.2(1.3;7.2)	4.7(0.1;9.4)	3.8(0.1;7.7)	2.1(−2.4;6.7)
	ADL	7.1(4.8;9.3)	5.0(1.7;8.3)	8.5(5.4;11.7)	−1.0(−4.5;2.5)
	Sport/Rec	4.5(1.3;7.6)	6.9(1.1;12.8)	2.4(0.8;5.6)	0.5(−4.4;5.4)
	QOL	5.8(3.1;8.6)	7.1(3.7;10.6)	4.8(0.7;9.0)	2.1 (−2.8;6.9)
**Physical function**					
Chair stands (sec)		−3.24(−4.29;-2.20)	−3.23(−4.33;-2.13)	−3.26(−4.96;-1.57)	−0.88(−1.33;-0.43)
Knee bendings/30 sec affected leg (n)^‡^		2.3(0.3;4.4)	4.0(1.4;6.7)	0.1(−3.1;3.2)	1.8(0.5;3.2)^†^
Knee bendings/30 sec non-affected leg (n)^‡^		1.4(−0.0;2.8)	2.3(−0.1;4.7)	0.6(−1.0;2.1)	
Knee ext affected leg (kg)		1.7(1.1;2.4)	1.9(0.9;2.7)	1.5(0.6;2.4)	0.2(−0.7;1.1)^†^
Knee ext non-affected leg (kg)		1.0(0.3;1.6)	1.2(0.1;2.3)	0.8(0.1;1.7)	
20-m walk test, time (sec)		−1.34(−1.97;-0.72)	−1.09(−1.85;-0.32)	−1.55(−2.51;-0.59)	−0.03(−0.32;0.26)
20-m walk test, steps (n)		−1.5(−2.3;-0.6)	−1.0(−1.9;-0.1)	−1.8(−3.1;-0.5)	−0.1(−0.5;0.3)

^†^Right leg in references used for comparison with patients’ affected and non-affected legs.

A negative value for chair stands and 20-m walk test (sec and steps) indicates improvement. For all other variables, a positive value indicates improvement.

^‡^Could not perform the test: Total OA group: affected leg n = 33, non-affected leg n = 15; Hip OA: affected leg n = 9, non-affected leg n = 4; Knee OA: affected leg n = 24, non-affected leg n = 11.

Ext = extension strength.

CI = confidence interval. Confidence intervals not containing zero indicate statistical significance.

### Effects of neuromuscular training

The mean number of training weeks was 11 (SD 6.1, range 2 to 34) for patients with hip OA and 13 (SD 5.1, range 4 to 28) for patients with knee OA (p = 0.251) (combined data, mean 12 weeks (SD 5.6, range 2 to 34)). The mean number of training sessions was 15 (SD 7.5, range 3 to 44) for patients with hip OA and 16 (SD 8.2, range 4 to 39) for those with knee OA (p = 0.366) (combined data, mean 16 training sessions (SD 7.9, range 3 to 44)).

Improvements by training were observed in all outcomes, except for number of knee bendings/30 seconds for the non-affected leg in patients with hip OA and for both the affected and non-affected legs in those with knee OA (Table 3).

The largest improvement in HOOS was noted for the subscale QOL and in KOOS for the subscale ADL (Table 3). The relative improvement ranged from median 9% (quartiles −5% to 29%) for HOOS ADL to median 29% (quartiles 0% to 100%) for HOOS Sport/Rec, and from median 7% (quartiles −7% to 30%) for KOOS Symptoms to 20% (quartiles −21% to 81%) for KOOS QOL.

**Figure 3:**
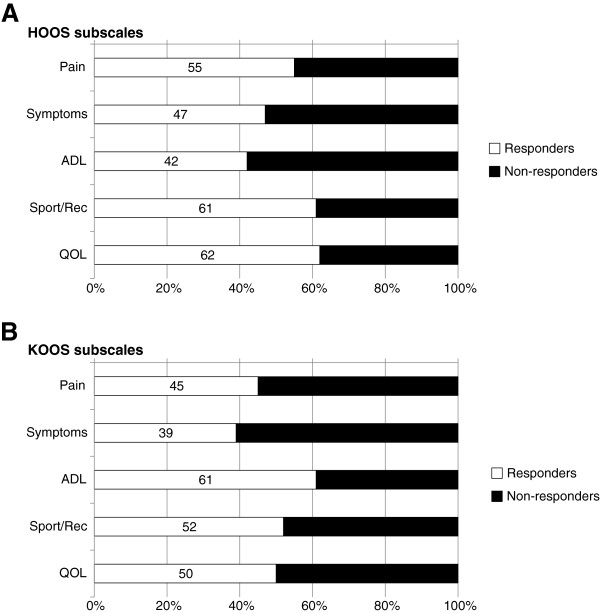
**Percentage of responders and non-responders for the five HOOS (A) or KOOS subscales (B).** An individual improving ≥15% was considered as responding to treatment.

Between 42% (HOOS ADL) and 62% (HOOS QOL) of the patients with hip OA, and between 39% (KOOS Symptoms) and 61% (KOOS ADL) of those with knee OA displayed an improvement ≥15% (responders) (Figure 3).

For physical function, the largest improvement was observed in chair stands, where both hip and knee OA patients performed the test about 3 seconds faster after the intervention (Table 3) (median improvement hip OA 18% (quartiles 8% to 24%), and knee OA 19% (quartiles 6% to 31%)). The smallest improvement was seen in number of steps for the 20-m walk test (median improvement hip OA 3% (quartiles 1% to 6%), knee OA 5% (quartiles −2% to 8%)).

### Influence of demographic factors on effects of training

Patients with hip OA showed greater improvements than those with knee OA for HOOS/KOOS subscale Sport/Rec and for the number of knee bendings in 30 seconds (Tables 4 and 5). Patients with knee OA displayed greater improvement than those with hip OA for HOOS/KOOS subscale ADL (Table 4).

**Table 4:** Influence of baseline demographic factors on change in patient-reported outcomes

		**HOOS/KOOS pain**	**HOOS/KOOS symptoms**	**HOOS/KOOS ADL**	**HOOS/KOOS sport/rec**	**HOOS/KOOS QOL**
		**Coefficient (95% CI)**	**Coefficient (95% CI)**	**Coefficient (95% CI)**	**Coefficient (95% CI)**	**Coefficient (95% CI)**
*Model*		*n = 87, R*^*2*^ *= 0.14*	*n = 87, R*^*2*^ *= 0.32*	*n = 85, R*^*2*^ *= 0.11*	*n = 83, R*^*2*^ *= 0.18*	*n = 86, R*^*2*^ *= 0.19*
	Joint (hip vs knee)	−0.5(−5.4;4.5)	−2.6(−8.3;3.0)	−4.1(−9.1;-0.8)	10.1(2.6;17.6)	3.9(−1.8;9.6)
	Sex (man vs woman)	0.4(−4.4;5.2)	−2.6(−8.2;3.0)	1.9(−2.9;6.7)	2.0(−4.5;8.5)	−2.2(−7.7;3.3)
	Age (y)	0.3(−0.3;0.8)	0.4(−0.3;1.0)	0.2(−0.4;0.8)	0.4(−0.4;1.1)	0.3(−0.3;1.0)
	BMI (kg/m^2^)	−0.2(−0.8;0.3)	−0.1(−0.7;0.6)	−0.1(−0.7;0.4)	0.0(−0.6;0.7)	−0.2(−0.9;0.4)
	Considered TJR (mths)	−0.0(−0.2;0.1)	−0.1(−0.2;0.0)	−0.0(−0.1;0.1)	−0.0(−0.2;0.1)	0.0(−0.1;0.2)
	Previous PT training (no vs yes)	5.1(−0.3;10.4)	9.7(3.6;15.7)	2.1(−3.2;7.4)	6.0(−1.1;13.3)	6.5(0.4;12.6)

All analyses adjusted for baseline HOOS/KOOS Pain. The corresponding baseline value adjusted for in the separate analyses.

CI = confidence interval. Confidence intervals not containing zero indicate statistical significance.

PT = Physical Therapy.

**Table 5:** Influence of baseline demographic factors on change in physical function measures

		**Chair stands (sec)**	**Knee bendings affected leg (n)**	**Knee bendings non-affected leg (n)**	**Knee ext affected leg (kg)**	**Knee ext non-affected leg (kg)**	**20-m walk test, time (sec)**	**20-m walk test, steps (n)**
		**Coefficient**	**Coefficient**	**Coefficient**	**Coefficient**	**Coefficient**	**Coefficient**	**Coefficient**
		**(95% CI)**	**(95% CI)**	**(95% CI)**	**(95% CI)**	**(95% CI)**	**(95% CI)**	**(95% CI)**
*Model*		*n = 86, R*^*2*^ *= 0.76*	*n = 47, R*^*2*^ *= 0.21*	*n = 66, R*^*2*^ *= 0.19*	*n = 86, R*^*2*^ *= 0.17*	*n = 86, R*^*2*^ *= 0.23*	*n = 86, R*^*2*^ *= 0.57*	*n = 86, R*^*2*^ *= 0.69*
	Joint (hip vs knee)	0.3(−0.9;1.5)	6.0(1.2;10.7)	3.3(0.3;6.4)	0.5(−0.9;1.9)	1.1(−0.3;2.5)	0.4(−0.5;1.4)	0.6(−0.4;1.6)
	Sex (man vs woman)	0.3(−0.9;1.4)	−1.3(−5.8;3.1)	−2.4(−5.4;0.7)	1.0(−0.6;2.5)	0.7(−0.8;2.3)	0.4(−0.6;1.3)	−0.7(−1.8;0.4)
	Age (y)	0.1(−0.1;0.2)	0.4(−0.2;1.1)	0.1(−0.2;0.5)	−0.2(−0.3;-0.1)	−0.1(−0.2;0.1)	−0.1(−0.2;0.1)	−0.1(−0.2;0.1)
	BMI (kg/m^2^)	0.0(−0.1;0.2)	−0.3(−0.9;0.3)	−0.2(−0.5;0.2)	0.1(−0.0;0.3)	0.3(0.2;0.5)	0.1(0.0;0.3)	0.2(0.1;0.3)
	Considered TJR (mths)	−0.1(−0.1;-0.0)	0.1(−0.2;0.3)	−0.0(−0.1;0.1)	−0.0(−0.0;0.0)	0.0(−0.0;0.0)	0.0(−0.0;0.0)	0.0(−0.0;0.0)
	Previous PT training (no vs yes)	−2.1(−3.4;-0.8)	0.8(−4.1;5.5)	1.4(−1.8;4.7)	0.6(−0.9;2.0)	0.6(−0.9;2.1)	−0.8(−1.8;0.3)	−1.1(−2.2;0.0)

All analyses adjusted for baseline HOOS/KOOS pain. The corresponding baseline value adjusted for in the separate analyses.

CI = confidence interval. Confidence intervals not containing zero indicate statistical significance.

PT = physical therapy.

Seventy-four percent of the patients reported no supervised or home training provided/instructed by a physical therapist within the last year before entering this study. This subgroup demonstrated greater improvements in HOOS/KOOS subscales symptoms and QOL, and in chair stands than those who reported that they had had such training (Tables 4, 5).

## Discussion

In this controlled before-and-after study, neuromuscular training showed promise for improving self-reported outcomes and physical function even in patients with severe primary OA of the hip or knee waiting for total joint replacement surgery. These findings should be confirmed in a randomized and controlled design.

There is limited research on the feasibility and benefits of neuromuscular exercise for people with OA. Most studies are small and include people with mild to moderate knee OA [17,33-35], and only two include patients with severe hip or knee OA [36,37]. These latter studies are small RCTs, evaluating the effect of pre-surgical training on post-surgical outcomes [36,37]. In one of these studies, within group changes by training were reported [36]. In patients with severe knee OA (n = 20) [36], “proprioceptive training” improved standing balance (unstable tilting platform device), while no effects were seen on patient-reported outcomes (WOMAC) or performance-based measures (gait speed, timed stairs test). In that study, no post-training data was provided for the control group (n = 15), thus, the change in outcome measures was not compared between groups [36]. Patients with severe hip OA [37], reported less pain and showed better standing balance (unstable tilting platform device) after a “sensorimotor training program” (n = 32) than a control group (n = 30) receiving no treatment, while no differences between groups were seen in patient-reported outcomes (SF-36, WOMAC). Because no data was provided for the outcomes at baseline [37], the within-group changes (before vs after training) observed in our study could not be compared with that study [37].

A small sample-size is a possible explanation for the few and small effects in these studies [36,37]. Factors related to the training intervention may also partly explain the limited effects. For example, focus on one specific aspect of sensorimotor function (standing balance in study [36]), improve primarily that function without necessarily affecting other aspects of sensorimotor function. Also, a standardized (not individualized) program without progression or supervision, as that described in [37], is in contrast to existing recommendations. Thus, an individualized approach to exercise with gradual progression [38], supervision [9], and exercises aiming at improving several aspects of sensorimotor function constitute essential components of training to sufficiently reduce symptoms and improve function.

Patients with knee or hip OA waiting for TJR report that reduced pain and improvements in activity/participation are equally important [25,26]. In the present study, mean pain was significantly but modestly reduced by approximately 6 points (median improvement 14%). HOOS/KOOS subscales ADL, Sport/Rec and QOL improved on average 7 (median 17%), 5 (median 20%), and 6 points (median 25%), respectively. Thus, neuromuscular training seemed effective for improving function and reducing pain. In the meta-analysis by Wallis et al. [3], modest reduction in pain was observed for hip and knee OA, but improvements in activity was noted for hip OA only, by aerobic exercise and/or strengthening training. A subject for further study is to elucidate whether neuromuscular training is more effective in improving outcomes than traditional treatment or usual exercise therapy.

Estimating improvement at the individual level is also important. Given that the minimal important change (MIC) appears to be dependent on the population and intervention, it may not be possible to define an MIC for a specific measurement instrument [39]. The MIC has not been established for KOOS/HOOS for patients with severe OA undergoing an exercise intervention. We applied the 15% responder criteria identified for exercise intervention in the WOMAC [31] to categorize an individual’s clinically meaningful improvement. The HOOS/KOOS subscale ADL is equivalent to the WOMAC subscale function. The 15% rather than the 50% responder criteria [40] was preferred since exercise is a low-cost intervention with few side effects [18]. Almost 50 percent of the patients demonstrated clinically meaningful reduction in pain and about 55 percent improved their function in ADL and sport/recreation. Taken together, the results at the group and individual levels suggest that the NEMEX-TJR program offers promise for a clinically relevant improvement in HOOS/KOOS by NEMEX-TJR.

The patients in the current study displayed improvements in performance-based measures; chair stands was performed about 3 seconds (median 19%) faster, and the 20-m walk test about 1 second (median 5%) faster, indicating a potential efficacy of neuromuscular training. Given that current research lacks the use of performance-based measures [3,6], the clinical relevance of improvements for groups and individuals is yet to be determined. About 38 percent of the patients were unable to perform the knee bending test on the affected leg, indicating that single-leg tests are challenging for these patients, at least before TJR. We included tests that are used by the OsteoArthritis Initiative (OAI) and have been proven reliable in people with severe hip or knee OA [27]. However, a standardized set of performance-based measures of physical function is yet to be determined [41].

Some demographic factors influenced change in outcomes. A possible reason for the differences between patients with hip and knee OA for change in HOOS/KOOS ADL and Sport/Rec may be the different anatomical characteristics and function of the joints. Nevertheless, these differences suggest that outcomes of hip or knee OA should be analyzed and reported separately. Having had no previous physical therapy training (supervised or home based) before entering the study was associated with greater improvements in HOOS/KOOS subscales symptoms and QOL, and in chair stands (adjusted for baseline value). This indicates an effect by training in addition to just having worse function at baseline.

Both self-reported and physical function measures were clearly worse in the patients compared with the reference group. The references HOOS/KOOS mean scores were somewhat better than age-specific KOOS scores in the adult population [42]. This was expected as we excluded those who had been treated for hip or knee disorders within the last year, in contrast to Paradowski et al. [42]. Thus, our reference data is likely appropriate for comparison with patients. The references were assessed twice, to account for any learning effect associated with the outcomes. The small systematic change observed for the chair stands and the number of knee bendings/30 seconds had no influence on the results, and was therefore not adjusted for in the analysis. Thus, the patients’ improvement from exercise may be interpreted as a real change. However, a randomized controlled study is required to rule out any potential biases of the current non-randomized design, such as placebo effect and regression towards the mean.

Not all eligible patients assigned for TJR were documented, and the severity of radiographic OA was not reported, denoting that a possible selection bias in our study cannot be excluded. However, our participants are comparable to other Scandinavian cohorts of patients with hip or knee OA awaiting TJR regarding sex-distribution [3], mean age [3], and HOOS/KOOS scores before surgery [25,26]. The patients who declined to participate in the supervised group training were on average 2.4 years younger than those who accepted participation, likely because more working-age individuals declined participation. Because this difference in age can be considered small, and since no differences were observed between participants and non-participants for any other demographic factors or baseline HOOS/KOOS, the risk of such selection bias is likely small.

## Conclusions

In conclusion, both self-reported and physical function measures were clearly worse compared with the reference group. Neuromuscular training showed promise for improving patient-reported outcomes and physical function measures, even in older patients with severe primary OA of the hip or knee before total joint replacement.

## Competing interests

The authors declare that they have no competing interests.

## Authors’ contributions

All authors contributed to the conception and design of the study. EA, AN, EMR contributed to acquisition of data. EA was responsible for analysis and interpretation of data, and drafted the manuscript. EMR contributed to data analysis and interpretation of data. All authors contributed to critical revision of the article for important intellectual content, and read and approved the final version.

## Pre-publication history

The pre-publication history for this paper can be accessed here:

http://www.biomedcentral.com/1471-2474/14/232/prepub

## References

[B1] Zhang W Nuki G Moskowitz RW Abramson S Altman RD Arden NK Bierma-Zeinstra S Brandt KD Croft P Doherty M OARSI recommendations for the management of hip and knee osteoarthritis: part III: changes in evidence following systematic cumulative update of research published through january 2009 Osteoarthritis Cartilage 2010 18 4 476 499 10.1016/j.joca.2010.01.013 20170770

[B2] Roos EM Juhl CB Osteoarthritis 2012 year in review: rehabilitation and outcomes Osteoarthritis Cartilage 2012 20 12 1477 1483 10.1016/j.joca.2012.08.028 22960093

[B3] Wallis JA Taylor NF Pre-operative interventions (non-surgical and non-pharmacological) for patients with hip or knee osteoarthritis awaiting joint replacement surgery - a systematic review and meta-analysis Osteoarthritis Cartilage 2011 19 12 1381 1395 10.1016/j.joca.2011.09.001 21959097

[B4] Mizner RL Petterson SC Clements KE Zeni JA Jr Irrgang JJ Snyder-Mackler L Measuring functional improvement after total knee arthroplasty requires both performance-based and patient-report assessments: a longitudinal analysis of outcomes J Arthroplasty 2011 26 5 728 737 10.1016/j.arth.2010.06.004 20851566 PMC3008304

[B5] Stratford PW Kennedy DM Riddle DL New study design evaluated the validity of measures to assess change after hip or knee arthroplasty J Clin Epidemiol 2009 62 3 347 352 10.1016/j.jclinepi.2008.06.008 18834709

[B6] Hoogeboom TJ van den Ende CH van der Sluis G Elings J Dronkers JJ Aiken AB van Meeteren NL The impact of waiting for total joint replacement on pain and functional status: a systematic review Osteoarthritis Cartilage 2009 17 11 1420 1427 10.1016/j.joca.2009.05.008 19500526

[B7] Roos EM Herzog W Block JA Bennell KL Muscle weakness, afferent sensory dysfunction and exercise in knee osteoarthritis Nat Rev Rheumatol 2011 7 1 57 63 10.1038/nrrheum.2010.195 21119605

[B8] Knoop J Steultjens MP van der Leeden M van der Esch M Thorstensson CA Roorda LD Lems WF Dekker J Proprioception in knee osteoarthritis: a narrative review Osteoarthritis Cartilage 2011 19 4 381 388 10.1016/j.joca.2011.01.003 21251988

[B9] Bennell KL Hunt MA Wrigley TV Lim BW Hinman RS Role of muscle in the genesis and management of knee osteoarthritis Rheum Dis Clin North Am 2008 34 3 731 754 10.1016/j.rdc.2008.05.005 18687280

[B10] Dekker J van Dijk GM Veenhof C Risk factors for functional decline in osteoarthritis of the hip or knee Curr Opin Rheumatol 2009 21 5 520 524 10.1097/BOR.0b013e32832e6eaa 19550331

[B11] Roos EM Joint injury causes knee osteoarthritis in young adults Curr Opin Rheumatol 2005 17 2 195 200 10.1097/01.bor.0000151406.64393.00 15711235

[B12] Zech A Hubscher M Vogt L Banzer W Hansel F Pfeifer K Neuromuscular training for rehabilitation of sports injuries: a systematic review Med Sci Sports Exerc 2009 41 10 1831 1841 10.1249/MSS.0b013e3181a3cf0d 19727032

[B13] Zätterström R Fridén T Lindstrand A Moritz U Muscle training in chronic anterior cruciate ligament insufficiency-a comparative study Scand J Rehabil Med 1992 24 2 91 97 1604267

[B14] Zätterström R Fridén T Lindstrand A Moritz U Early rehabilitation of acute anterior cruciate ligament injury-a randomized clinical trial Scand J Med Sci Sports 1998 8 3 154 159 9659676 10.1111/j.1600-0838.1998.tb00186.x

[B15] Ericsson YB Dahlberg LE Roos EM Effects of functional exercise training on performance and muscle strength after meniscectomy: a randomized trial Scand J Med Sci Sports 2009 19 2 156 165 18397193 10.1111/j.1600-0838.2008.00794.x

[B16] Ageberg E Consequences of a ligament injury on neuromuscular function and relevance to rehabilitation-using the anterior cruciate ligament-injured knee as model J Electromyogr Kinesiol 2002 12 3 205 212 10.1016/S1050-6411(02)00022-6 12086815

[B17] Stensrud S Roos EM Risberg MA A 12-week exercise therapy program in middle-aged patients with degenerative meniscus tears: a case series with 1-year follow-up J Orthop Sports Phys Ther 2012 42 11 919 931 22960783 10.2519/jospt.2012.4165

[B18] Ageberg E Link A Roos EM Feasibility of neuromuscular training in patients with severe hip or knee OA: the individualized goal-based NEMEX-TJR training program BMC Musculoskelet Disord 2010 11 126 10.1186/1471-2474-11-126 20565735 PMC2896351

[B19] Peat G McCarney R Croft P Knee pain and osteoarthritis in older adults: a review of community burden and current use of primary health care Ann Rheum Dis 2001 60 2 91 97 10.1136/ard.60.2.91 11156538 PMC1753462

[B20] Carr AJ Robertsson O Graves S Price AJ Arden NK Judge A Beard DJ Knee replacement Lancet 2012 379 9823 1331 1340 10.1016/S0140-6736(11)60752-6 22398175

[B21] Manjer J Carlsson S Elmståhl S Gullberg B Janzon L Lindström M Mattisson I Berglund G The malmo diet and cancer study: representativity, cancer incidence and mortality in participants and non-participants Eur J Cancer Prev 2001 10 6 489 499 10.1097/00008469-200112000-00003 11916347

[B22] Williams GN Chmielewski T Rudolph K Buchanan TS Snyder-Mackler L Dynamic knee stability: current theory and implications for clinicians and scientists J Orthop Sports Phys Ther 2001 31 10 546 566 11665743 10.2519/jospt.2001.31.10.546

[B23] Thomee R A comprehensive treatment approach for patellofemoral pain syndrome in young women Phys Ther 1997 77 12 1690 1703 9413448 10.1093/ptj/77.12.1690

[B24] Roos EM Roos HP Lohmander LS Ekdahl C Beynnon BD Knee injury and osteoarthritis outcome score (KOOS)–development of a self-administered outcome measure J Orthop Sports Phys Ther 1998 28 2 88 96 9699158 10.2519/jospt.1998.28.2.88

[B25] Nilsdotter AK Lohmander LS Klassbo M Roos EM Hip disability and osteoarthritis outcome score (HOOS)–validity and responsiveness in total hip replacement BMC Musculoskelet Disord 2003 4 10 10.1186/1471-2474-4-10 12777182 PMC161815

[B26] Roos EM Toksvig-Larsen S Knee injury and osteoarthritis outcome score (KOOS) - validation and comparison to the WOMAC in total knee replacement Health Qual Life Outcomes 2003 1 17 10.1186/1477-7525-1-17 12801417 PMC161802

[B27] Villadsen A Roos EM Overgaard S Holsgaard-Larsen A Agreement and reliability of functional performance and muscle power in patients with advanced osteoarthritis of the Hip or knee Am J Phys Med Rehabil 2012 91 5 401 10 10.1097/PHM.0b013e3182465ed0 22311054

[B28] Bremander AB Dahl LL Roos EM Validity and reliability of functional performance tests in meniscectomized patients with or without knee osteoarthritis Scand J Med Sci Sports 2007 17 2 120 127 17394472 10.1111/j.1600-0838.2006.00544.x

[B29] Andrews AW Thomas MW Bohannon RW Normative values for isometric muscle force measurements obtained with hand-held dynamometers Phys Ther 1996 76 3 248 259 8602410 10.1093/ptj/76.3.248

[B30] Martin HJ Yule V Syddall HE Dennison EM Cooper C Aihie Sayer A Is hand-held dynamometry useful for the measurement of quadriceps strength in older people? a comparison with the gold standard bodex dynamometry Gerontology 2006 52 3 154 159 10.1159/000091824 16645295

[B31] Hurley MV Walsh NE Mitchell H Nicholas J Patel A Long-term outcomes and costs of an integrated rehabilitation program for chronic knee pain: a pragmatic, cluster randomized, controlled trial Arthritis Care Res (Hoboken) 2012 64 2 238 247 10.1002/acr.20642 21954131

[B32] Angst F Aeschlimann A Stucki G Smallest detectable and minimal clinically important differences of rehabilitation intervention with their implications for required sample sizes using WOMAC and SF-36 quality of life measurement instruments in patients with osteoarthritis of the lower extremities Arthritis Rheum 2001 45 4 384 391 10.1002/1529-0131(200108)45:4<384::AID-ART352>3.0.CO;2-0 11501727

[B33] Bennell KL Egerton T Wrigley TV Hodges PW Hunt M Roos EM Kyriakides M Metcalf B Forbes A Ageberg E Comparison of neuromuscular and quadriceps strengthening exercise in the treatment of varus malaligned knees with medial knee osteoarthritis: a randomised controlled trial protocol BMC Musculoskelet Disord 2011 12 276 10.1186/1471-2474-12-276 22141334 PMC3247187

[B34] Thorstensson CA Henriksson M von Porat A Sjödahl C Roos EM The effect of eight weeks of exercise on knee adduction moment in early knee osteoarthritis–a pilot study Osteoarthritis Cartilage 2007 15 10 1163 1170 10.1016/j.joca.2007.03.012 17466541

[B35] Smith TO King JJ Hing CB The effectiveness of proprioceptive-based exercise for osteoarthritis of the knee: a systematic review and meta-analysis Rheumatol Int 2012 32 11 3339 3351 10.1007/s00296-012-2480-7 22821333

[B36] Gstoettner M Raschner C Dirnberger E Leimser H Krismer M Preoperative proprioceptive training in patients with total knee arthroplasty Knee 2011 18 4 265 270 10.1016/j.knee.2010.05.012 20801047

[B37] Bitterli R Sieben JM Hartmann M de Bruin ED Pre-surgical sensorimotor training for patients undergoing total hip replacement: a randomised controlled trial Int J Sports Med 2011 32 9 725 732 10.1055/s-0031-1271696 21630176

[B38] Roddy E Zhang W Doherty M Arden NK Barlow J Birrell F Carr A Chakravarty K Dickson J Hay E Evidence-based recommendations for the role of exercise in the management of osteoarthritis of the hip or knee–the MOVE consensus Rheumatology (Oxford) 2005 44 1 67 73 10.1093/rheumatology/keh399 15353613

[B39] Terwee CB Roorda LD Dekker J Bierma-Zeinstra SM Peat G Jordan KP Croft P de Vet HC Mind the MIC: large variation among populations and methods J Clin Epidemiol 2010 63 5 524 534 10.1016/j.jclinepi.2009.08.010 19926446

[B40] Pham T van der Heijde D Altman RD Anderson JJ Bellamy N Hochberg M Simon L Strand V Woodworth T Dougados M OMERACT-OARSI initiative: osteoarthritis research society international set of responder criteria for osteoarthritis clinical trials revisited Osteoarthritis Cartilage 2004 12 5 389 399 10.1016/j.joca.2004.02.001 15094138

[B41] Dobson F Hinman RS Hall M Terwee CB Roos EM Bennell KL Measurement properties of performance-based measures to assess physical function in hip and knee osteoarthritis: a systematic review Osteoarthritis Cartilage 2012 20 12 1548 1562 10.1016/j.joca.2012.08.015 22944525

[B42] Paradowski PT Bergman S Sunden-Lundius A Lohmander LS Roos EM Knee complaints vary with age and gender in the adult population. Population-based reference data for the knee injury and osteoarthritis outcome score (KOOS) BMC Musculoskelet Disord 2006 7 38 10.1186/1471-2474-7-38 16670005 PMC1471783

